# Supplementary material for: A Machine Learning Approach to Determine Risk Factors for Respiratory Bacterial/Fungal Coinfection in Critically Ill Patients with Influenza and SARS-CoV-2 Infection: A Spanish Perspective
Source: Antibiotics (Basel). 2024 Oct 14;13(10):968. doi: 10.3390/antibiotics13100968 (PMC11504409; doi:10.3390/antibiotics13100968)

A Machine Learning approach to determine risk factors for respiratory Bacterial/Fungal co-infection in critically ill patients with influenza and COVID-19: a Spanish perspective

Supplementary material

Figure S1: Flow chart of patients included in the analysis

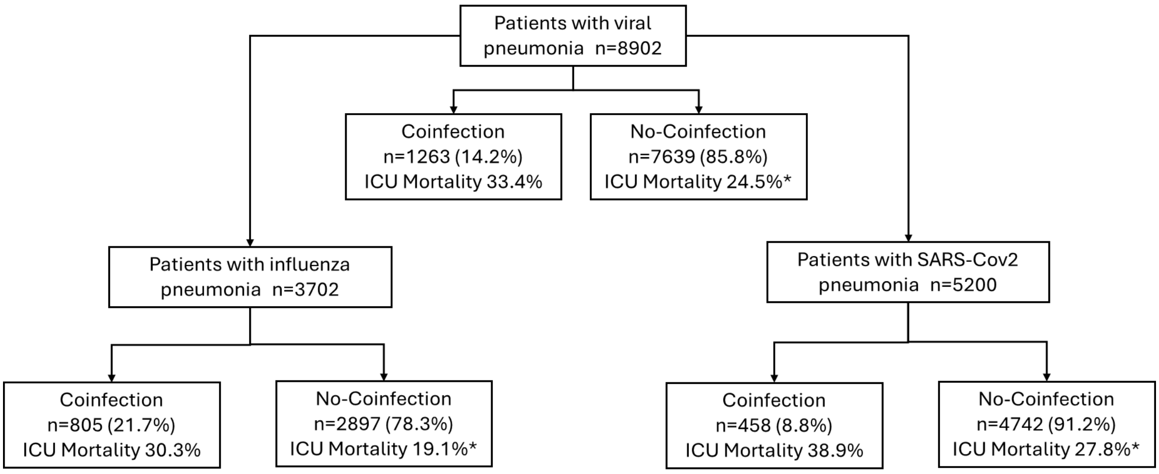

GLM Model

Table S1: Performance of predictive GLM model for coinfection

Point estimates and 95% CIs:

Apparent prevalence *	0.14 (0.13, 0.16)
True prevalence *	0.37 (0.35, 0.39)
Sensitivity *	0.23 (0.20, 0.26)
Specificity *	0.91 (0.89, 0.92)
Positive predictive value *	0.60 (0.55, 0.65)
Negative predictive value *	0.67 (0.65, 0.69)
Positive likelihood ratio	2.53 (2.09, 3.06)
Negative likelihood ratio	0.85 (0.82, 0.88)
False T+ proportion for true D- *	0.09 (0.08, 0.11)
False T- proportion for true D+ *	0.77 (0.74, 0.80)
False T+ proportion for T+ *	0.40 (0.35, 0.45)
False T- proportion for T- *	0.33 (0.31, 0.35)
Correctly classified proportion *	0.66 (0.64, 0.68)

* Exact CIs

Figure S2: Discrimination (AUC) of the GLM model for predicting coinfection

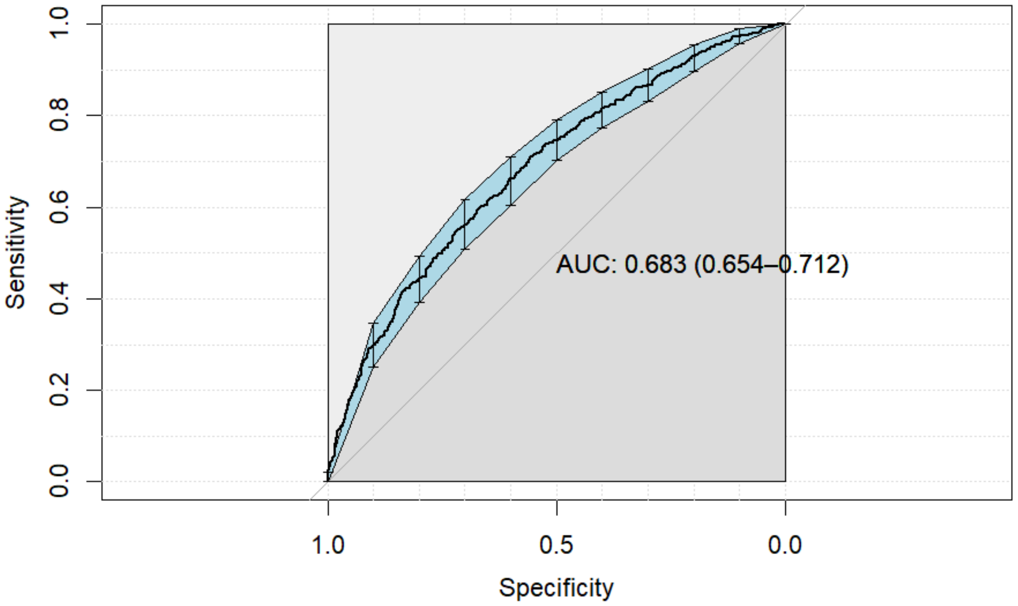

Table S2: Cross-validation (K=10) of the GLM model to predict the presence of co-infection.

```
Confusion Matrix and Statistics

          Reference
Prediction    0    1
         0 1530  155
         1  762  224

               Accuracy : 0.6567
                 95% CI : (0.6383, 0.6747)
    No Information Rate : 0.8581
    P-Value [Acc > NIR] : 1

                  Kappa : 0.155

 Mcnemar's Test P-Value : <2e-16

            Sensitivity : 0.6675
            Specificity : 0.5910
         Pos Pred Value : 0.9080
         Neg Pred Value : 0.2272
             Prevalence : 0.8581
         Detection Rate : 0.5728
   Detection Prevalence : 0.6308
      Balanced Accuracy : 0.6293

       'Positive' Class : 0
```

Figure S3: Residual plot distribution of overall model

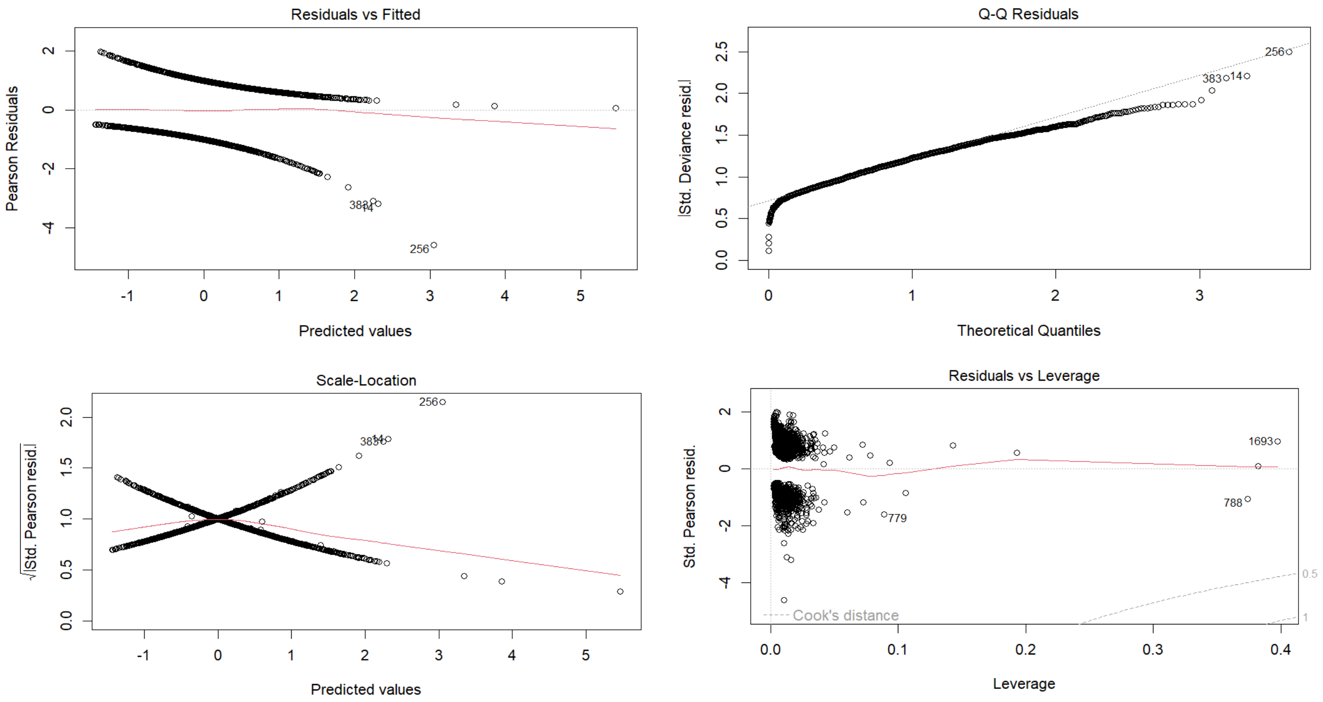

Figure S4: Variables associated with presence of coinfection in patients with influenza in the GLM model.

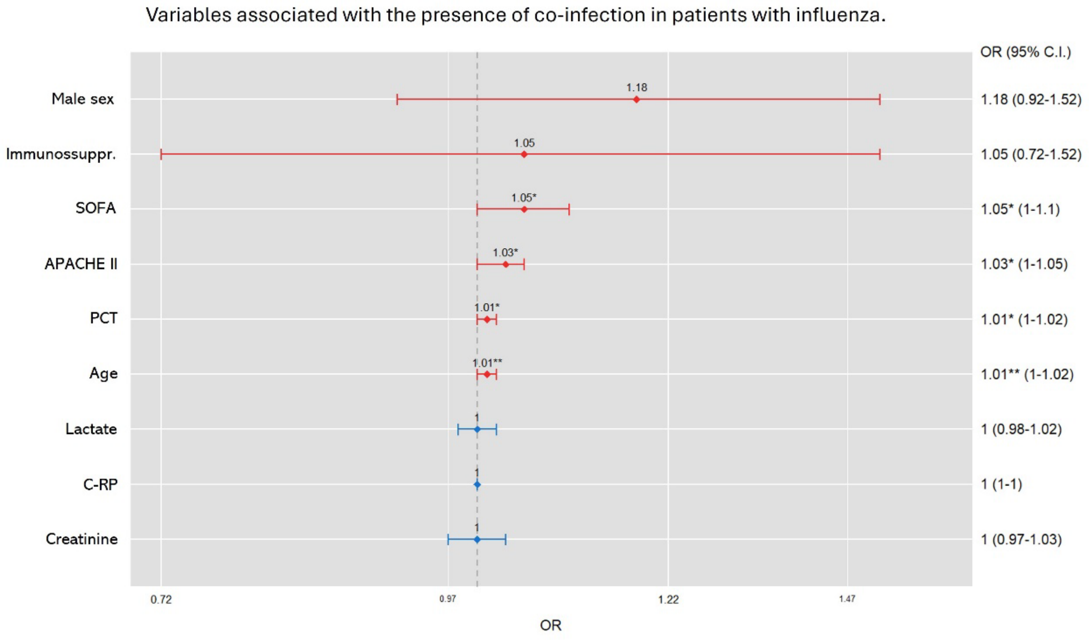

Table S3: Performance of predictive GLM model for coinfection in influenza patients

```
Point estimates and 95% CIs:
--------------------------------------------------------------
Apparent prevalence *                  0.22 (0.19, 0.24)
True prevalence *                      0.46 (0.43, 0.49)
Sensitivity *                          0.31 (0.27, 0.35)
Specificity *                          0.86 (0.83, 0.89)
Positive predictive value *            0.66 (0.60, 0.72)
Negative predictive value *            0.60 (0.56, 0.63)
Positive likelihood ratio              2.29 (1.80, 2.91)
Negative likelihood ratio              0.80 (0.74, 0.85)
False T+ proportion for true D- *      0.14 (0.11, 0.17)
False T- proportion for true D+ *      0.69 (0.65, 0.73)
False T+ proportion for T+ *           0.34 (0.28, 0.40)
False T- proportion for T- *           0.40 (0.37, 0.44)
Correctly classified proportion *      0.61 (0.58, 0.64)
--------------------------------------------------------------
* Exact CIs
```

Figure S5: Discrimination (AUC) of the GLM model for predicting coinfection in influenza patients.

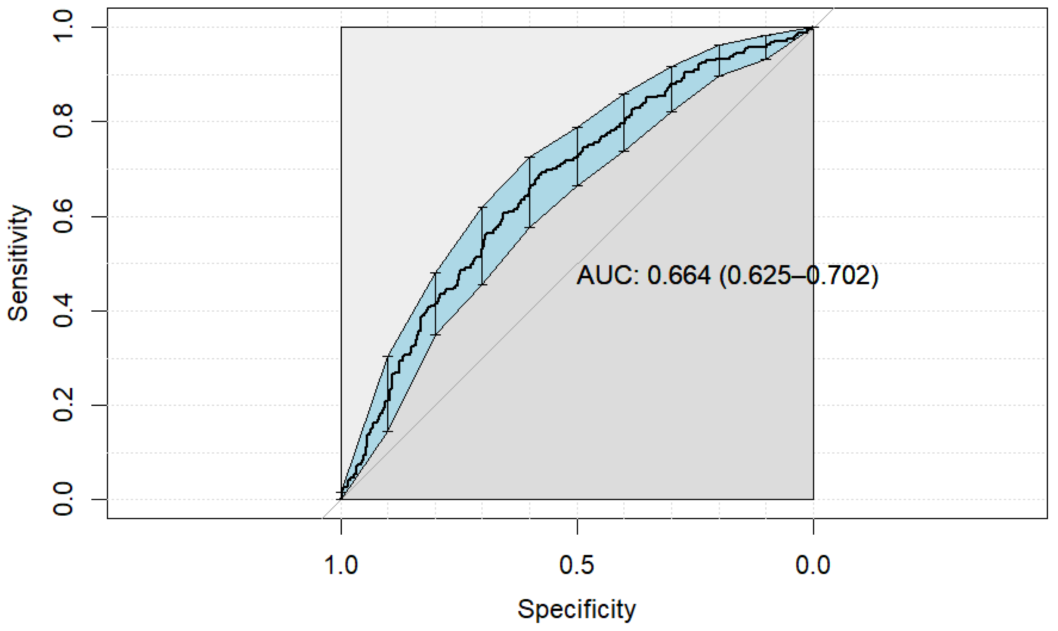

Table S4: Cross-validation (K=10) of the GLM model to predict the presence of co-infection in influenza patients

```
Confusion Matrix and Statistics

          Reference
Prediction   0   1
         0 500  85
         1 369 156

               Accuracy : 0.591
                 95% CI : (0.5614, 0.6201)
    No Information Rate : 0.7829
    P-Value [Acc > NIR] : 1

                  Kappa : 0.1562

 Mcnemar's Test P-Value : <2e-16

            Sensitivity : 0.5754
            Specificity : 0.6473
         Pos Pred Value : 0.8547
         Neg Pred Value : 0.2971
             Prevalence : 0.7829
         Detection Rate : 0.4505
   Detection Prevalence : 0.5270
      Balanced Accuracy : 0.6113

       'Positive' Class : 0
```

Figure S6: Plot of important variables of the Random Forest classifier model in influenza patients

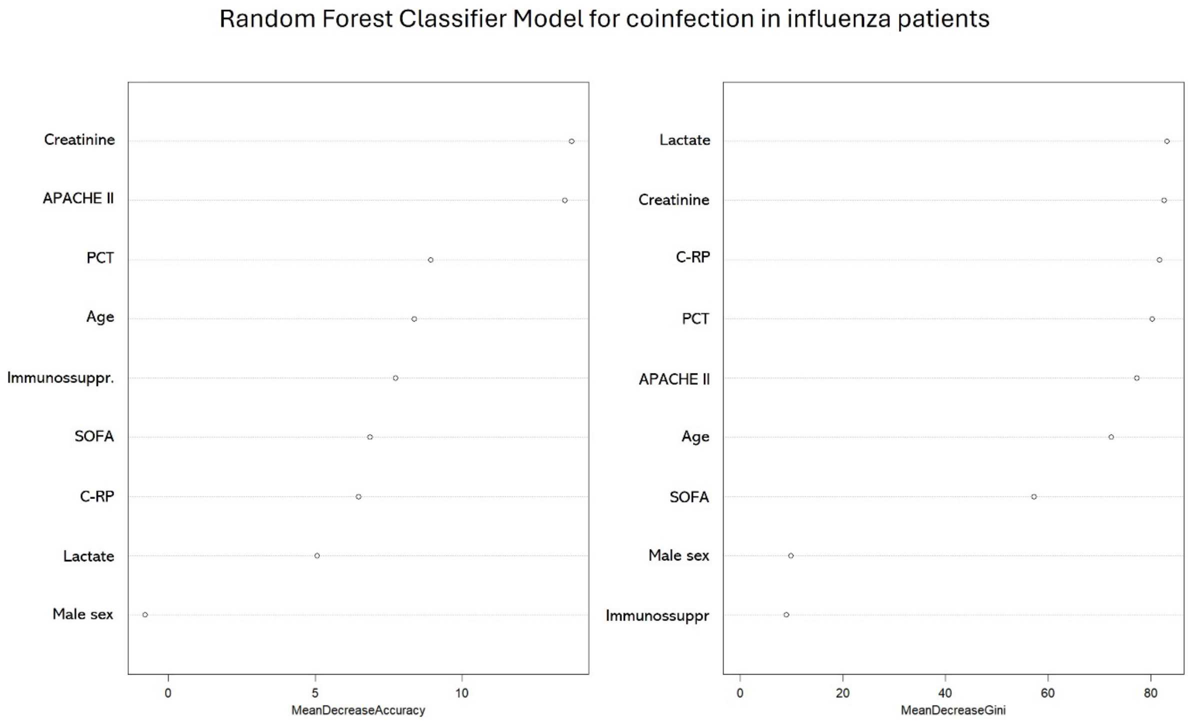

Figure S7: Variables associated with presence of coinfection in patients with COVID-19 in the GLM model.

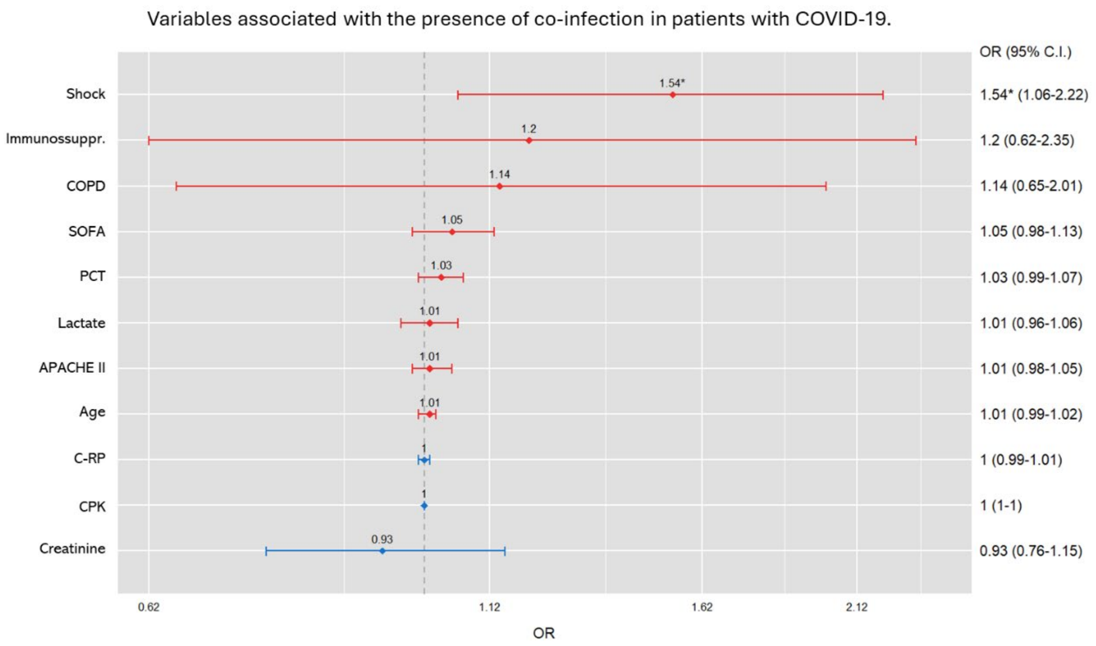

Table S5: Performance of predictive GLM model for coinfection in COVID-19 patients

Point estimates and 95% CIs:

Apparent prevalence *	0.09 (0.07, 0.10)
True prevalence *	0.42 (0.40, 0.45)
Sensitivity *	0.11 (0.08, 0.13)
Specificity *	0.92 (0.91, 0.94)
Positive predictive value *	0.50 (0.42, 0.59)
Negative predictive value *	0.59 (0.56, 0.61)
Positive likelihood ratio	1.39 (1.01, 1.92)
Negative likelihood ratio	0.97 (0.94, 1.00)
False T+ proportion for true D- *	0.08 (0.06, 0.09)
False T- proportion for true D+ *	0.89 (0.87, 0.92)
False T+ proportion for T+ *	0.50 (0.41, 0.58)
False T- proportion for T- *	0.41 (0.39, 0.44)
Correctly classified proportion *	0.58 (0.55, 0.60)

* Exact CIs

Figure S8: Discrimination (AUC) of the GLM model for predicting coinfection in COVID-19 patients.

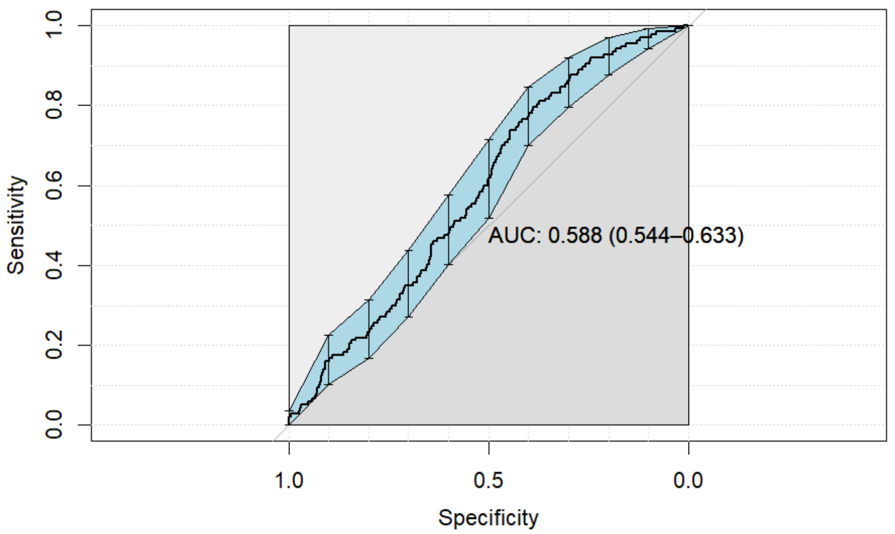

Table S6: Cross-validation (K=10) of the GLM model to predict the presence of co-infection in COVID-19 patients

```
Confusion Matrix and Statistics

          Reference
Prediction   0   1
         0 684  41
         1 739  96

               Accuracy : 0.5
                 95% CI : (0.4749, 0.5251)
    No Information Rate : 0.9122
    P-Value [Acc > NIR] : 1

                  Kappa : 0.0549

 Mcnemar's Test P-Value : <2e-16

            Sensitivity : 0.4807
            Specificity : 0.7007
         Pos Pred Value : 0.9434
         Neg Pred Value : 0.1150
             Prevalence : 0.9122
         Detection Rate : 0.4385
   Detection Prevalence : 0.4647
      Balanced Accuracy : 0.5907

       'Positive' Class : 0
```

Figure S9: Plot of important variables of the Random Forest Classifier model (COVID-19)

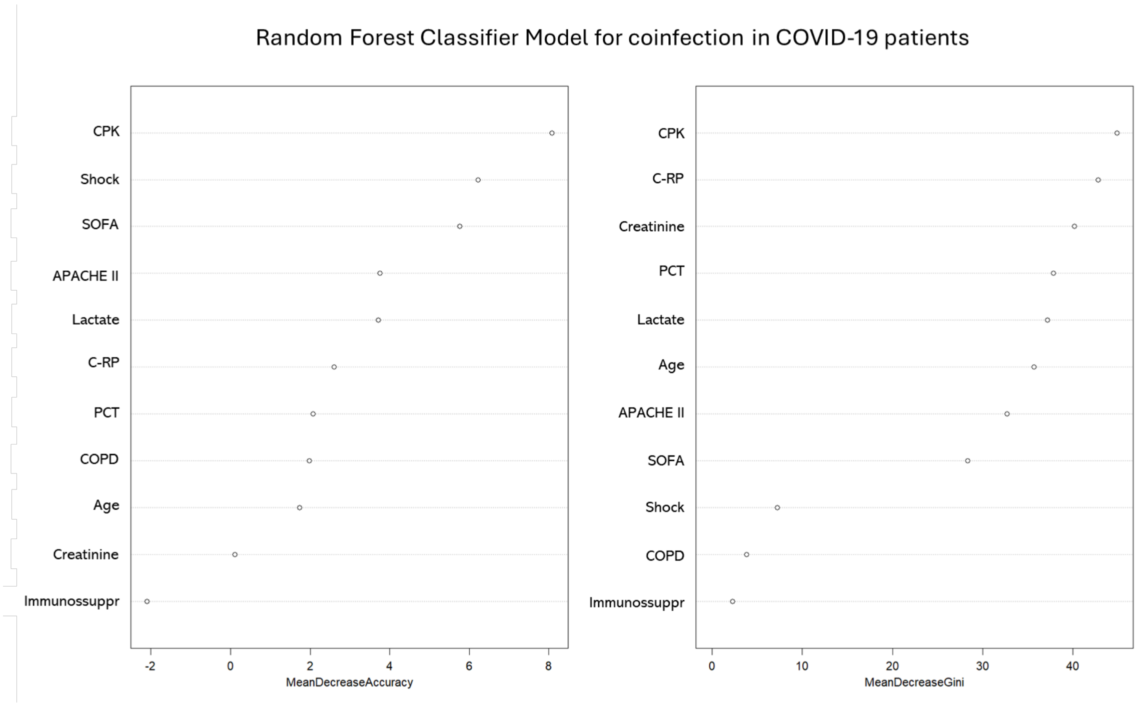

Supplement: Supplementary file 1 [file antibiotics-13-00968-s001.zip › antibiotics-3233520-supplementary.pdf]
